# Fundamental roles of reactive oxygen species and protective mechanisms in the female reproductive system

**DOI:** 10.1186/1477-7827-3-43

**Published:** 2005-09-02

**Authors:** Junichi Fujii, Yoshihito Iuchi, Futoshi Okada

**Affiliations:** 1 Department of Biomolecular Function, Yamagata University Graduate School of Medicine, 2-2-2 Iidanishi, Yamagata 990-9585, Japan

## Abstract

Controlled oxidation, such as disulfide bond formation in sperm nuclei and during ovulation, plays a fundamental role in mammalian reproduction. Excess oxidation, however, causes oxidative stress, resulting in the dysfunction of the reproductive process. Antioxidation reactions that reduce the levels of reactive oxygen species are of prime importance in reproductive systems in maintaining the quality of gametes and support reproduction. While anti-oxidative enzymes, such as superoxide dismutase and peroxidase, play a central role in eliminating oxidative stress, reduction-oxidation (redox) systems, comprised of mainly glutathione and thioredoxin, function to reduce the levels of oxidized molecules. Aldo-keto reductase, using NADPH as an electron donor, detoxifies carbonyl compounds resulting from the oxidation of lipids and proteins. Thus, many antioxidative and redox enzyme genes are expressed and aggressively protect gametes and embryos in reproductive systems.

## Introduction

Since reproductive and developmental process accompany dynamic changes in metabolism and energy consumption, byproducts are also generated on an extraordinary scale. Among such byproducts, reactive oxygen species (ROS), which are inevitably generated during the physiological process of oxygen consumption, the levels of which are enhanced under some pathological conditions [1], are the most troublesome. Although ROS as well as nitric oxide (NO), which is produced in a limited amount in response to physiological stimuli, are considered to mediate inter- and intra-cellular signaling, generation of an excess them results in oxidative stress, which is determined by the balance between oxidants and antioxidants, and becomes the primary or secondary cause in deterioration, due to various diseases. Gametes are extremely sensitive to damage by ROS and must be protected against to maintain the species. Low molecular weight compounds, such as antioxidative vitamins (A, C, and E) and glutathione, react with ROS and convert them to harmless compounds. In addition, living organisms have evolved enzymatic systems that effectively suppress oxidative stress and minimize damage caused by ROS.

It is, however, not possible to eliminate all ROS and, hence, oxidative modification occurs in the building blocks of cells. Oxidative stress leads to disulfide bond formation of sulfoxidation in sulfhydryl residues in proteins. Unsaturated lipids are prone to oxidation and are converted to peroxides, a target of the peroxidase reaction, followed by the generation of degradation products with an aldehyde moiety. The resulting aldehyde compounds are more or less toxic and must be detoxified. Small molecules can be simply discarded into the urine, but the remainder must be reduced by a corresponding reduction-oxidation (redox) system. However, contents of carbonyl groups in oxidized proteins are generally increased and considered to be a marker of the oxidative modification of proteins and aging [2]. Thus, the reduction potential of NAD(P)H is of prime importance in the maintenance of the redox balance of cells. Nucleic acids suffer from oxidative modification, with the base moiety being the preferred target. However, in most cases, damaged DNA can be efficiently repaired by several systems according to the double stranded nature of DNA. Since unrepaired bases are mutagenic, cells carrying them must also be eliminated.

The physiological relevance of antioxidative/redox systems in male reproductive tract has been overviewed recently [3] and, hence, are only briefly mentioned here. The emphasis of this review is on systems in female reproductive organs under physiological and pathological conditions and viewpoint is extended to fertilization and early embryonal development.

## Origin of oxidative, nitrosative, and carbonyl stress

ROS are generated via various reactions in the body. Some ROS are produced by non-enzymatic reactions, for example via the Fenton reaction in the presence of transient metal ions [1]. Biological reactions, such as electron transfer and oxygenase reactions that utilize oxygen molecules as the substrate, also generate large amounts of ROS. Since the mitochondrial respiratory chain is the main oxygen-consuming system in cells, the majority of ROS are produced from this system under physiological conditions. The generation of ROS becomes excessive under conditions of elevated metabolism and pathological conditions and is matter of special concern. The following are well-known enzymatic systems that generate ROS. Xanthine dehydrogenase, an enzyme involved in purine metabolism, is converted to xanthine oxidase that generates superoxide under ischemic conditions in cardiovascular systems [4]. Cyclooxygenase, which catalyzes the initial oxidation step in the conversion from arachidonate to prostanoids and is induced under inflammatory conditions, also generates ROS [5]. The generation of ROS by the P450 system is important during the metabolic process of steroid hormone synthesis from cholesterol in endocrine organs, such as the ovary and testis. Professional phagocytes, such as neutrophils, contain NADPH-oxidase that generates huge amounts of superoxide for microbicidal purposes.

Nitrogen oxide species (RNOS) are mainly derived from nitric oxide (NO) and also play various roles in reproductive organs [6]. Nitric oxide synthase (NOS), which is encoded by three different genes, NOS I, NOS II, and NOS III, catalyzes the formation of NO from arginine and oxygen using NADPH as an electron donor. Among NO derived from the three isoforms of NOS, NO from nNOS (NOS I) appears to function as a neurotransmitter. NO generated from endothelial NOS (NOS III) is involved in vascular relaxation. NOS III expression is increased by luteinizing hormone (LH) surge or human chorionic gonadotropin (hCG). NOS III may also be involved in oocyte maturation and the ovulatory process as described below. The expression of inducible NOS (NOS II) is induced by many stimuli, including inflammatory cytokines. Since a certain fraction of NO is converted to harmful RNOS, such as peroxynitrite by reaction with ROS, nitrosative stress occurs simultaneously with ROS generation. NOS II is also commonly a matter of concern in reproductive organs under pathological conditions because it produces large amounts of NO in response to inflammatory and other stimuli.

Malondialdehyde and 4-hydroxy-2-nonenal levels are increased under conditions of oxidative stress and are still toxic because they carry aldehyde group. Carbonyl compounds are produced under hyperglycemic conditions, in addition to oxidative and nitrosative stress, and increase during the aging process. Amino acid residues in proteins are also subject to oxidative modification and are converted into carbonyl containing compounds. Since carbonyl compounds are also reactive toward thiols and amino groups and cause carbonyl stress [7], detoxification by reduction constitutes another pivotal system.

## Antioxide/redox system

Many low molecular weight antioxidants, such as antioxidative vitamins and polyphenols, are ordinarily present in nutrients. Although ROS are scavenged by these compounds, enzymatic detoxification is more efficient [1]. The following major antioxidative enzymes are present in our body.

### Superoxide dismutase (SOD)

The superoxide anion is produced by a one-electron reduction of an oxygen molecule and initiates a radical chain reaction. It is believed that SOD, which dismutates the superoxide anion to hydrogen peroxide, plays a central part in antioxidative reactions. Three isozymes are produced by mammalians.

SOD1 encodes Cu,Zn-SOD that contains Cu and Zn as metal cofactors and is largely cytosolic, while SOD2-encoding Mn-SOD is a mitochondrial isoform containing Mn. SOD3, which encodes the extracellular form (EC-SOD), is structurally similar to CuZn-SOD, and also contains Cu and Zn as metal cofactors. Since a mutation in SOD1 causes amyotrophic lateral sclerosis, extensive studies have been carried out in neuronal cells. One of the striking phenotypes of SOD1-deficient mice is female infertility and this is discussed below.

Mn-SOD is a mitochondrial isoform but its gene, SOD2, is encoded by nuclear DNA. SOD2 is inducible under various oxidative stress and inflammatory conditions and, hence, the regulatory mechanism of the gene has been a subject of extensive study. Homozygous SOD2-deficient mice suffer severe cardiovascular damage and die soon after birth [8]. Although no abnormality in the genital tract has been reported for heterozygous mice, transgenic male mice that express higher levels of Mn-SOD are infertile but the mechanism for this is unknown [9].

EC-SOD is present at high levels in the epididymis as well as the lung [10]. EC-SOD is also localized in the nuclei in the seminiferous tubules of the testis [11]. Superoxide decreases levels of NO by converting it to peroxinitrite. Thus, scavenging superoxide in vasculature extends the half-life of nitric oxide (NO), which results in an increase in cGMP levels. It is probable that elevated levels of cGMP relax vascular smooth muscle and supports erectile responses. Erectile function is improved by transferring the SOD3 gene to the penis in aged rats [12]. However, no recognizable phenotype in the reproductive system has yet been reported in SOD3 knockout mice [13].

### Peroxidases

Glutathione peroxidase (GPx) plays a central role in the detoxification of peroxides using the reduced form of glutathione (GSH) as an electron donor. Many enzymes that are classified into different family of proteins exhibit GSH-dependent peroxidase activity. Conventional GPx contains selenocysteine (Sec) at its active center and appears to play a pivotal role in detoxification of peroxides. At least four selenium-containing GPx isozymes are produced in mammalians. The cytosolic form, GPX1, is widely distributed in tissues and has been the most extensively investigated form. However, GPX1-knockout mice show no abnormality in phenotype including reproductive capability [14]. GPX2 encodes a gastrointestinal form, and no specific function for it is known in reproduction. GPX3 is present in plasma and epididymal fluid. GPX4 encodes an isoform that specifically detoxifies phospholipid hydroperoxide is thus referred to as PhGPx, and is expressed at high levels in the testis. A defect in GPX4 has been suspected as a cause of male infertility triggered by Se deficiency, although direct evidence for its requirement is missing [15]. GPX4 protein represents about 50% of the capsule material that embeds the helix of mitochondria in the midpiece of spermatozoa [16]. A correlation between male infertility and a GPX4 defect has actually been reported [17,18]. Thus, GPX4 may have some physiological role in the male reproductive system. A novel isoform is specifically present in sperm nuclei and is considered to act as a protamine thiol peroxidase. Since the molecule has a high sequence identity to GPX4, except for the N-terminal region [19], it is likely that they are products of the same gene and are generated by an alternate promoter and exon usage of GPX4 [20].

Catalase exclusively detoxifies hydrogen peroxide and has no requirement for an electron donor. It plays a role in organs such as the liver, but its specific function in the genital tract is largely unknown. The number of enzymes carrying peroxidase activity is still increasing. Peroxiredoxins are recently identified multifunctional redox proteins with peroxidase activity that require electrons from thioredoxin [21].

### Glutathione redox system

**Figure 1:**
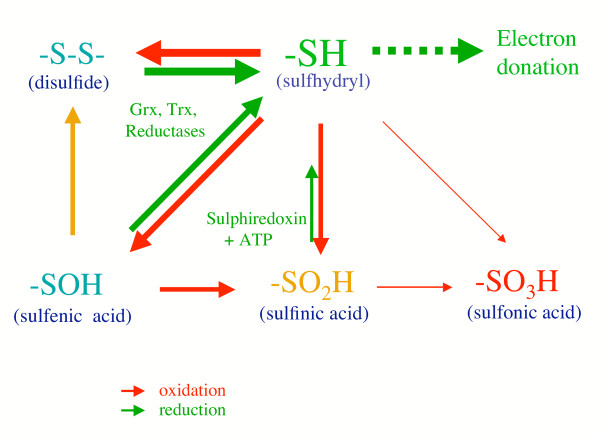
Reaction of sulfhydryl group in response to oxidative stress and interconversion among the oxidation products. Sulfhydryl residues form different oxidative states that largely depends on the source and extent of oxidative stress. Since the redox system generally relies on reactive sulfhydryls, whether they are reversible or not is of prime importance.

The sulfhydryl residue forms various redox states, as illustrated in Figure 1. Mild sulfhydryl oxidation produces disulfides and sulfenic acids, which are easily converted to disulfides by reaction with an adjacent sulfhydryl residue. Sulfenic acid is further oxidized to sulfinic acid and then to sulfonic acid. Disulfides and sulfenic acids are reduced back to the sulfhydryl stage by thioredoxin (Trx), glutatredoxin (Grx), and other thiol reductases under high redox potential. Recent reports have shown that sulfinic acid also can be reduced back to the sulfhydryl stage although the reaction requires ATP and, hence, is not a simple reduction reaction [22]. Sulfonic acid is not reversibly reduced to a sulfhydryl under physiological conditions. It is not possible to evaluate their generation in cells accurately because they are highly reactive and in a dynamic equilibrium.

Glutathione is a tripeptidyl molecule and is present in either the reduced (GSH) or the oxidized state (GSSG) by forming a disulfide bond between two molecules. It has pleiotropic roles, which include the maintenance of cells in a reduced state and the formation of conjugates with some harmful endogenous and xenobiotic compounds [23]. In addition, GSH serves as an electron donor for glutathione peroxidase that reduces peroxide to the corresponding alcohol, as described above. GSH levels are maintained by *de novo *synthesis that is catalyzed by two enzymes, γ-glutamylcysteine synthetase (γ-GCS) and glutathione synthetase (GS). The rate-limiting step in glutathione synthesis is the first reaction, in which γ-glutamylcysteine is formed, catalyzed by -GCS. An increase in GSH levels in response to various stimuli is mainly attributed to the responsiveness of -GCS gene expression to the stimuli. Buthionine sulfoximine (BSO), a specific inhibitor for γ-GCS, is thus commonly used to deplete intracellular GSH. The reduction of GSSG is catalyzed by glutathione reductase (GR) using NADPH as an electron donor. Nitrosourea (BCNU), an anti-cancer drug, is used to inhibit GR [24]. One of anti-cancer functions of BCNU is, therefore, attributable to the inhibition of GR and the lowering of GSH levels. GR is also inhibited by compounds produced in response to nitrosative stress, such as nitrosoglutathione. In the female reproductive system, GSH is assumed to play a role in reducing oxidative stress either by interaction directly with ROS or by donating electron to GPx.

### Thioredoxin (Trx) system

Trx, originally identified as an electron donor for ribonucleotide reductase, functions to regulate various enzymes and trans-activating factors of genes, and is intimately involved in cell growth, differentiation, and death [25]. Trx also functions as a protein disulfide isomerase that corrects disulfide bridges that are formed in error. Moreover, Trx directly donates electrons to peroxiredoxin and, hence, is directly linked to the peroxidase reaction [21]. After oxidation, an intramolecular disulfide bond is formed in Trx. Oxidized thioredoxin is reduced by thioredoxin reductase, a selenocystine-containing oxido-reductase, using NADPH as an electron donor. Since Trx-knockout mice are embryonically lethal [26], Trx appears to play essential roles in the reproductive system and/or fetal development. Among multiple roles of Trx, defect in electron donation to ribonucleotide reductase appears to be the main cause in Trx-knockout mice because DNA synthesis is essential for fetal development.

### Aldo-keto reductase

Carbonyl compounds are produced by the oxidation of organic compounds, such as unsaturated fatty acids, and are highly reactive. They modify reactive sulfhydryl groups that are commonly present in redox-sensitive molecules, resulting in an impairment of the systems. Mammalians have several enzymatic systems that function to detoxify carbonyl compounds. The aldo-keto reductase family includes enzymes that reduce carbonyl groups to alcohol using NADPH as an electron donor. Among the members of this family, aldose reductase, the *AKR1B *gene product, has been the most extensively studied because it is intimately involved in diabetic complications [27]. An inhibitor of aldose reductase is one of proposed cures for diabetic complications. Aldehyde reductase, the *AKR1A *gene product, exhibits the highest similarity to aldose reductase among the family members [28] and appears to play a coordinate function [29]. Since steroid hormones and their derivatives contain carbonyl groups and can serve as substrates for aldo-keto reductase [30], enzymes that are highly expressed in tissues with steroid hormone production may have a role in their elimination. The detoxification of carbonyls is activated by the binding of GSH, which indicates crosstalk between the GSH redox system and aldo-keto reductase system [31].

## Physiological relevance of ROS/RNOS and antioxide/redox enzymes in female reproductive system

### ROS and antioxidative system

**Figure 2:**
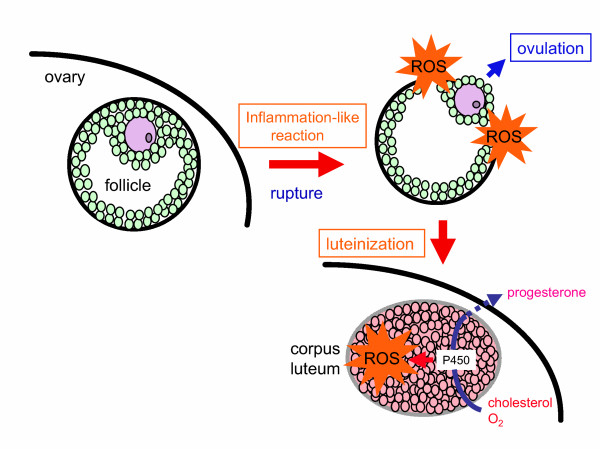
Generation of ROS during ovulation and sterodogenesis in corpus luteum. Ovulation appears to be an inflammation-like process. ROS is locally produced during follicular rupture and may be involved in the ovulation process. ROS is also generated by the corpus luteum via the monooxygenase reaction as a byproduct during steroid hormone synthesis.

Ovary is a metabolically active organ and, hence, is under a variety of stresses continuously. ROS play a physiological role during ovulation that is similar in some respects to inflammation [32,33]. Ovulation is suppressed by agents that inhibit acute inflammatory reactions [34]. Since ROS is generated during inflammatory process, it is reasonably hypothesized that ROS is released in connection with follicle rupture and is involved in the process (Figure 2). The source of ROS appears to be inflammatory cells, such as macrophages and neutrophils, as they are present in ovary at ovulation [35-37] and produce tremendous amount of free radical. This notion is supported by the finding that the suppression of ROS by SOD and/or catalase in in-vitro perfused rabbit ovary preparations hinders ovulation [38]. Monooxygenase reaction, mediated by P450, is required for the steroidogenic process that inevitably produces ROS as byproducts. ROS levels in the corpus luteum actually increase during the regression phase [39-44]. The NADPH-dependent generation of superoxide in the mouse ovary increases during the early pre-ovulatory phase in cycling females and during the luteal phase in pregnant animals [45]. Ovarian as well as uterine NADPH-dependent superoxide production appears to be LH-inducible. ROS and related compounds may function as intracellular regulators of steroidogenesis and progesterone release in the corpus luteum [41,46-48].

SOD is present in growing follicles, the membrane granulosa of Graafian follicles, ovulated follicles, and blood vessels. Cyclic changes in SOD levels during the reproductive cycle of rats and an inverse correlation between the levels of SOD and superoxide radical have been reported [49]. SOD may play a role in regulating follicular development, ovulation, and luteal functions [50]. In the gestational corpus luteum, theca and granulosa lutein cells show strong and moderate staining intensity, respectively [51]. SOD activity is also present in human pre-ovulatory ovarian follicular fluid at higher levels than in serum [52]. About a 7-fold higher level of SOD activity is present in porcine follicular fluid and appears to exert protection against oxidative damage in oocytes [53].

SOD levels are controlled by several humoral factors and vice versa. Gonadotropoin-mediated rat follicular development coincides with an enhanced expression of Mn-SOD and EC-SOD mRNA [54]. Mn-SOD expression is induced and suppresses apoptosis in the rabbit corpus luteum in vitro, suggesting that Mn-SOD is responsible for the gonadotropin-mediated inhibition of apoptosis [55]. Both CuZn-SOD and Mn-SOD mRNA level are increased in the rat corpus luteum by prolactin [56]. However, Cu,Zn-SOD and Mn-SOD are differently regulated by estrogen and progesterone in human endometrial stromal cells. The decrease in Cu,Zn-SOD after ovarian steroid withdrawal may be involved in endometrial breakdown [57]. In case of Mn-SOD, estrogen withdrawal led to an enhanced expression of TNF-α [58], which would increase Mn-SOD mRNA levels and Mn-SOD activity in a dose-dependent manner in human endometrial stromal cells [59]. The decrease in Cu,Zn-SOD expression and the increase in lipid peroxide in the decidua may be involved in the termination of spontaneous abortion, which is mediated via the increase in PGF2α synthesis. This suggests that Cu,Zn-SOD contributes to the maintenance of pregnancy by preventing the accumulation of superoxide radicals that causes PGF2α synthesis [60]. The stimulation of luteal Cu,Zn-SOD expression by HCG may be important in maintaining luteal cell integrity when pregnancy occurs [61]. In the process of decidualization, estradiol plus medroxyprogesterone acetate increases Mn-SOD expression via a cAMP-dependent pathway. Cu,Zn-SOD is also up-regulated by these compounds, but via a different pathway from that involving cAMP [62].

### Roles of redox system

In addition to antioxidation, redox systems are also well developed and protect organs against damage by oxidative stress. GSH synthesis by cumulus cells occurs during *in vitro *oocyte maturation in cows [63,64] and during *in vivo *meiotic maturation in hamsters [65,66]. Oocytes, granulosa cells, and lutein cells all express high levels of GR [67]. Because the corpus luteum produces much of the progesterone in conjunction with the reaction of P450s by consuming molecular oxygen and, hence, produces ROS as a byproduct, damage could be inflicted by ROS. Cumulus cells participate in the enhancement of GSH content in oocytes and the protection of oocytes against oxidative stress-induced apoptosis [68]. The detoxification of the produced ROS by GSH in conjunction with antioxidative enzymes would be particularly important for the corpus luteum and surrounding cells.

GSH is present in oviductal fluids and may be involved in development of mouse embryos [69]. The high levels of GR in the epithelia of the oviducts would account for this finding [67]. The secreted GSH would protect oocytes against excessively produced ROS that occurs during the ovulation, thus maintaining fertilization potency. Many *in vitro *studies indicate significance of antioxidants for oocyte maturation and embryo development [e.g. [70,71]].

ROS and, in consequence, carbonyl compounds can be produced by activated metabolism. Thus, detoxification by aldo-keto reductase appears to contribute to the maintenance of the genital tract. In fact, granullosa cells and the epithelia of the genital tract produce high levels of aldose reductase and aldehyde reductase [72]. The separate role of these enzymes in maintaining reproductive function is a matter of concern. Aldose reductase is an enzyme that reduces carbonyls including steroid metabolites [31] to the corresponding alcohols. It is known that aldose reductase is hormonally regulated in rat ovary during the estrous cycle [73].

### Roles of RNOS

RNOS also plays multiple roles in the ovary [6]. Of the three NOS isozymes, NOS II and NOS III are expressed in the ovary [74-77]. The expression of NOS III increases after a LH surge or hCG injection. The expression of NOS III in oocytes and the blockade of oocyte maturation by the oral administration of NOS inhibitors have been reported [78]. NO generated from NOS III stimulates the ovulatory process [79-84]. Oocyte meiotic maturation is arrested in NOS III knockout mice [83,85]. However, the source of NO as it relates to oocyte maturation is currently under debate. NOS II is mainly localized in granulosa cells and produces large amounts of NO. The decrease in nitrate/nitrite concentration in preovulatory follicles after a hCG injection is correlated mainly to a decreased NOS II expression in granulosa cells [86]. NO, generated from NOS that is present in human granulosa-luteal cells, appears to inhibit estradiol secretion by directly inhibiting aromatase [87]. In addition, excess NO inhibits progesterone production and causes apoptotic cell death in rat granulose cells [86,88]. An NO-donor, *S*-nitroso-*N*-acetyl-D,L-penicillamine (SNAP), dose-dependently inhibits germinal vesicle break down in denuded oocytes, and this effect of SNAP can be reversed by the addition of hemoglobin [74]. These data suggest that the NOS II-NO-(cGMP) system may play a role in oocyte meiotic maturation, but further studies will be required to ascertain the actual function in the ovary.

## ROS and RNOS in fertilization and early development of embryo

**Figure 3:**
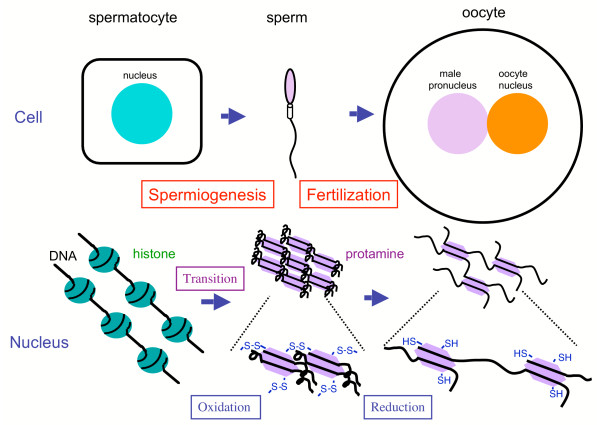
Redox regulation of spermatogenesis and fertilization. During the spermatogenic process, histones are converted to protamines via transition proteins in sperm nuclei. The maturation of spermatozoa proceeds in the epididymis. Oxidation mediated by sulfoxidase is involved in the packaging of chromatin into the small nucleus via disulfide bridge formation between protamines. After fertilization, the sperm head expands to the male pronucleus by reducing the disulfide bond in the oocyte.

The deteriorating effects of the oxidation reaction in sperm cells have been generally discussed and overviewed [89,90]. However, oxidation reactions, in conjunction with the appropriate redox system, also exert beneficial roles. One of the most striking functions is sulfoxidation in sperm nuclei during their maturation. While ROS easily damages DNA, the regulated oxidation of sulfhydryls to disulfide in protamines is required for sperm maturation in the epididymis (Figure 3) [91]. The regulated sulfoxidation plays a role in the correct packaging of the nucleus into the small sperm head and resistance to ROS during the fertilization process, GPX3 and GPX4 present in the epididymal fluid may be responsible for the reduction of coincidently produced peroxides [92]. After fertilization, a high redox potential is required for male pronuclear formation by reducing disulfide bonds in oocytes. The origin of the reducing power appears to be GSH in the nucleus [93] because GSH present at 9–10 mM is the major source of redox potential in the oocyte [94,95]. Oocytes are also rich in glutathione reductase [69] and support the view. Since GSH alone is not effective in the reduction of disulfide bonds, cross-reactions with Trx, which has protein disulfide isomerase activity, may occur. Glycolytic activity, which generates NADH, and the hexose monophosphate shunt, a regenerating system for NADPH, are enhanced during the penetration of spermatozoa into oocytes [96], and an elevated redox potential appears to be involved in fertilization [97]. Reducing equivalents generated during the conversion of 3α-androstanediol to 5α-dihydrotestosterone has been proposed to be an alternate source of NAD(P)H [98]. The coordinate activation of these redox and NAD(P)H generating systems would enable early embryonic development to proceed.

Peri-hatching blastocysts generate a considerably large amount of ROS for an extremely short period of time when compared to unhatched and hatched blastocysts [99]. Despite the potential importance of SOD1, knockout mice are born and grow normally. The most striking phenotype is the infertility of the SOD1-deficient female [100,101]. In spite of a precise examination, the actual cause of embryonic lethality is unknown [100]. Although both homozygous and heterozygous embryos grow normally in heterozygous females, both embryos die in homo-knockout females. This suggests the cause can be attributed to a maternal factor. Other groups have reported defects in ovary function in homo-knockout mice [101]. Since SOD is involved in the elimination of superoxide that is generated during steroidogenesis, this may be related to steroidogenesis in the ovary. Among mice that are deficient in other antioxidative enzymes, GPX4-knockout mice show premature embryonal death in the uteri, but the direct cause of this is also not clear [102,103]. A comparative study of both knockout mice may provide a clue to understanding the mechanism.

The glutathione redox system is also deeply involved in embryogenesis. Preimplanted embryos are very sensitive to conditions that cause oxidative stress. Their glutathione status changes dramatically during development [104]. GSH in reproductive tract fluid may help protect preimplanted embryos from the adverse effects of toxicants [68]. Usefulness of glutathione in embryo production has been demonstrated in culture system [105]. Increased embryonic fragmentation and a slow cleavage rate may be partially attributed to the early exposure of embryos to high ROS levels in intracytoplasmic sperm injection cycles [106]. The presence of BSO decreases GSH levels to a greater extent in the blastocyst than in the two-cell embryo [107]. GSH synthesis and turnover increase between the two-cell and blastocyst stages. The increase in the ability of embryos to synthesize GSH on day 3 is dependent on protein synthesis [108]. Hence, the recycling of GSSG must play an important role in maintenance of intracellular GSH levels from the oocyte to the two-cell stage.

The placenta is rich in aldose reductase. The presence of abundant aldose reductase in uterine luminal fluids and term placenta has been detected by two-dimensional gel electrophoresis [109]. Although aldose reductase in conjunction with sorbitol dehydrogenase catalyzes the conversion of glucose to fructose, which can be the energy source for the spermatozoa [110], the quantity of aldose reductase appears to be in excess. Both enzymes are abundant in eggs and may participate in the production of fructose [72]. Thus, aldose reductase appears to have additional roles, beyond detoxification. The production of certain cytokines, such as IL-1 and TNF, are elevated and, hence, ROS levels are also elevated. The detoxification of carbonyls present in reproductive tract fluids would be advantageous to embryos at their early developmental stage in oviducts and the uterus. Steroid metabolites such as isocorticosteoids and progesterone [30] and lipid peroxidation products such as 4-hydroxynonenal and acrolein [111,112] are all aldose reductase substrates. The glutathione conjugate of 4-hydroxy-3-nonenal actually serves as a substrate for aldose redcutase [113]. Glutathione S-transferase is known to be present in the reproductive system [114], and, hence, the presence of GSH could facilitate the detoxification function of aldose reductase by producing glutathione conjugates. The production of carbonyl compounds is caused mainly by ROS, the level of which increases during repoduction processes such as cell proliferation, steoidgenesis, and ovulation.

## Conclusion

Since the production of ROS is high in reproductive tissue due to active metabolism and steroidogenesis, the tissue is under continuous oxidative stress. ROS modifies susceptible molecules including DNA, lipids, and proteins. Carrying such damage in oocytes increases the risk of hereditable disease, and, hence, living organisms must eliminate such gametes to preserve the species. On the other hand, the reproductive system utilizes ROS in some processes that are essential for reproduction. To minimize the risk caused by ROS, antioxidative systems, such as SOD and GPX have been developed. When ROS levels exceed the scavenging capacity of the system, a redox system, under such situations, can repair oxidized and damaged molecules using NADPH as an original electron source. Thus, the maintenance of a high redox potential is prerequisite for maintaining the reproductive systems in a healthy state.
